# Self-reported contacts for mental health problems by rural residents: predicted service needs, facilitators and barriers

**DOI:** 10.1186/s12888-014-0249-0

**Published:** 2014-09-06

**Authors:** Tonelle E Handley, Frances J Kay-Lambkin, Kerry J Inder, Terry J Lewin, John R Attia, Jeffrey Fuller, David Perkins, Clare Coleman, Natasha Weaver, Brian J Kelly

**Affiliations:** Centre for Translational Neuroscience and Mental Health, University of Newcastle and Hunter New England Health, Callaghan, NSW Australia; Centre for Rural and Remote Mental Health, University of Newcastle, Orange, NSW Australia; National Drug and Alcohol Research Centre, University of New South Wales, Sydney, NSW Australia; Hunter Medical Research Institute, Locked Bag 1, Hunter Region Mail Centre, Newcastle, NSW Australia; Centre for Clinical Epidemiology and Biostatistics, University of Newcastle and Hunter New England Health, Newcastle, NSW Australia; School of Nursing and Midwifery, Flinders University, Adelaide, SA Australia

**Keywords:** Rural, Mental health, Service utilisation, Treatment barriers, Attitudes

## Abstract

**Background:**

Rural and remote Australians face a range of barriers to mental health care, potentially limiting the extent to which current services and support networks may provide assistance. This paper examines self-reported mental health problems and contacts during the last 12 months, and explores cross-sectional associations between potential facilitators/barriers and professional and non-professional help-seeking, while taking into account expected associations with socio-demographic and health-related factors.

**Methods:**

During the 3-year follow-up of the Australian Rural Mental Health Study (ARMHS) a self-report survey was completed by adult rural residents (N = 1,231; 61% female; 77% married; 22% remote location; mean age = 59 years), which examined socio-demographic characteristics, current health status factors, predicted service needs, self-reported professional and non-professional contacts for mental health problems in the last 12 months, other aspects of help-seeking, and perceived barriers.

**Results:**

Professional contacts for mental health problems were reported by 18% of the sample (including 14% reporting General Practitioner contacts), while non-professional contacts were reported by 16% (including 14% reporting discussions with family/friends). Perceived barriers to health care fell under the domains of structural (e.g., costs, distance), attitudinal (e.g., stigma concerns, confidentiality), and time commitments. Participants with 12-month mental health problems who reported their needs as met had the highest levels of service use. Hierarchical logistic regressions revealed a dose-response relationship between the level of predicted need and the likelihood of reporting professional and non-professional contacts, together with associations with socio-demographic characteristics (e.g., gender, relationships, and financial circumstances), suicidal ideation, and attitudinal factors, but not geographical remoteness.

**Conclusions:**

Rates of self-reported mental health problems were consistent with baseline findings, including higher rural contact rates with General Practitioners. Structural barriers displayed mixed associations with help-seeking, while attitudinal barriers were consistently associated with lower service contacts. Developing appropriate interventions that address perceptions of mental illness and attitudes towards help-seeking is likely to be vital in optimising treatment access and mental health outcomes in rural areas.

**Electronic supplementary material:**

The online version of this article (doi:10.1186/s12888-014-0249-0) contains supplementary material, which is available to authorized users.

## Background

International evidence suggests that the prevalence of mental health (MH) problems is similar across rural and urban areas [1,2]. In Australia, although mental illness may occur at similar rates across geographical regions [3,4], rural areas generally witness considerably lower MH service use [5,6]. Distance to services is among the most significant issues [7], with longer travelling time to MH services associated with clients making fewer visits, and being less likely to receive care in adherence with treatment guidelines [8]. The shortage of local services (especially specialist services) also contributes to longer waiting periods, which has been identified by rural General Practitioners (GP) as a referral deterrent [9]. Residing a greater distance from urban centres is associated with fewer community health centre contacts, outpatient services and days spent in inpatient wards [10].

Previous research has found that even where MH services are available, people residing in rural areas display lower help-seeking [6,11]. This may be due to attitudes which value self-reliance and a preference for self-management of MH problems, as well as higher stigma in rural areas [11,12]. In fact, perceived stigma may be more central in determining MH service use than even the severity of the condition or the level of disability [13,14]. Conversely, positive attitudes towards help-seeking, and believing that a GP would be helpful for MH concerns, are significantly associated with lifetime GP consultations by rural residents, after controlling for current psychological distress [15]. Confidentiality is also an important issue, with rural residents expressing concerns that their personal information may be disclosed, or that other residents will see them attending healthcare services [16].

There is some evidence that negative attitudes about treatment effectiveness are higher in more remote areas of Australia [17] and mixed evidence about the contribution of perceived stigma towards mental illness [13,15,18]. Simultaneously, increasing remoteness is typically associated with greater difficulties accessing services [19] and potentially greater reliance on internet-derived information [20]. Due to potential correlations between these distinct “barriers types” (i.e., attitudinal, physical/structural barriers), it is necessary to examine both constructs in order to establish their relative impact in relation to help-seeking. This has been explored previously, with findings indicating a greater influence of attitudinal than structural barriers [21–23]. However, past research has generally focused on urban populations, which may differ in their patterns of structural and attitudinal barriers. Understanding the relative contributions of different barriers to rural MH service use is vital to ensure that efforts to optimise service use are appropriately directed.

Our earlier report from the Australian Rural Mental Health Study (ARMHS) examined baseline cross-sectional associations between socio-demographic, current health status, and service utilization variables [24]; however, at that time, limited data had been collected about service use barriers. The observed baseline rate of professional contacts for MH problems during the previous 12 months exceeded the national rate (17% *vs*. 11.9%) [25]. This was primarily due to higher rural contact rates with GPs (12% *vs*. 8.1%), which may largely reflect the older age of the ARMHS sample. Selected data from the ARMHS 3-year follow-up have also been examined [26] to try to better understand the feasibility of using internet-delivered MH treatments among rural populations, together with relevant barriers (e.g., internet availability, treatment acceptability, previous exposure).

The current analysis of the ARMHS 3-year follow-up data is a more comprehensive exploration of perceived barriers to MH care in rural areas, and their cross-sectional associations with professional and non-professional help-seeking. Importantly, this analysis seeks to account for the influence of socio-demographic factors, including geographical remoteness, and current health status factors, thereby enabling a more thorough exploration of the role of perceived structural, attitudinal and other barriers. In short, the primary questions of interest for this paper are: 1) who self-reports current MH problems and associated help-seeking; 2) what potential facilitators/barriers can be identified; and 3) how much of a contribution do these facilitators/barriers make to reported professional and non-professional help-seeking, after taking into account the influence of more traditional indicators (e.g., socio-demographic, location, and health related factors). In broad terms, it was hypothesised that there would be a dose-response relationship between predicted service needs and reported rates of service use (i.e., higher service usage among those with higher severity of problems) and that perceived barriers, particularly attitudinal barriers, would be associated with lower reported service usage.

## Methods

### Study sample

Participants included in the current analysis completed the third phase (3-year follow-up) of the ARMHS project, a longitudinal investigation of MH in rural and remote New South Wales (NSW). Baseline data collection began in May 2007, with follow-ups conducted at 1-, 3- and 5-years post-baseline; the 3-year follow-up was conducted from February 2011 to March 2012. Residents of non-metropolitan areas were selected randomly from the Electoral Roll, with recruitment covering four Australian Standard Geographical Classification (ASGC) categories: inner regional, outer regional, remote, and very remote. Deliberate over-sampling from remote and very remote regions was undertaken to ensure sufficient representation from these areas. See Kelly et al. [27,28] for full descriptions of the ARMHS project and sampling methods.

Written informed consent was obtained from each participant with the return of their baseline postal survey. Ethical approval was obtained from the Human Research Ethics Committees of the Universities of Newcastle and Sydney, and the Greater Western, Hunter New England and North Coast Area Health Services.

### Measures

#### Participant characteristics

Socio-demographic items included questions about age, gender, marital status, and commitments, including regular employment, living with children, and caring for a disabled or ill family member/friend. Perceived financial circumstances was rated on a 6-point scale from ‘prosperous’ to ‘very poor’, adapted from the Household, Income and Labour Dynamics in Australia (HILDA) Survey [29].

#### Predicted need for MH services

Each individual’s likely current need for professional MH services was quantified using the Predicted Service Need Index (PSNI). The PSNI was initially developed using baseline ARMHS survey data [as detailed in 24], with the integrity of the scoring algorithms re-confirmed using 3-year survey data; see Additional file 1 for further details. Overall scores on the PSNI range from 0 to 14, obtained by summing the integer weights (from 0 to 3) assigned to 16 categories across seven health status measures: overall ratings of mental and physical health; Kessler-10 (K10) [2,30]: current psychological distress; Alcohol Use Disorders Identification Test (AUDIT) [31,32]: current hazardous alcohol use; Patient Health Questionnaire-9 (PHQ-9) [33]: current depressive symptoms; recent adverse life events; and current smoking status. In addition to its use as a continuous measure (with higher scores reflecting an increased likelihood or predicted need for professional services), three PSNI categories have also been identified: *low* (0–1); *medium* (2–5); and *high* (>5) estimated need; see earlier report [24] and Additional file 1 for further details.

#### Suicidal ideation

An independent measure of suicidal ideation was also included; item 9 of the PHQ-9 asks respondents to rate the frequency of “thoughts that you would be better off dead, or of hurting yourself in some way” during the past two weeks; responses were dichotomised into a ‘yes/no’ variable.

#### MH problems, contacts and barriers

The trigger question for the section of the 3-year follow-up survey enquiring about recent MH problems and contacts was: “In the past 12 months have you experienced any *mental health problems* such as stress, anxiety or depression or worries about alcohol or drugs?” Thereafter, participants with a positive response to this question were asked several questions regarding their help-seeking during the previous 12-months, including: a) whether they had sought any help or advice for MH problems; b) where they sought help (and how many times); c) the type of help received (e.g., information about MH, medication); and d) whether they felt they had received as much help as needed. For the 3-year follow-up, a scannable booklet was used, necessitating some changes to the response options and layout (relative to earlier phases). The combined questions about sources and frequency of contacts included 11 professional sources (e.g., GP, psychiatrist, psychologist, MH nurse, Lifeline, specialist doctor), and 4 non-professional sources (e.g., family/friends, alternative therapist, clergy). Seven response options were provided for the frequency of contacts in the last 12 months with each source, ranging from ‘None’, ‘1–2 times’, to ‘13+ times’, which were assigned weights of 0, 2, 4, 6, 9, 12, and 18 contacts respectively.

Participants with self-identified MH problems during the last 12 months who either: a) did not seek help; or b) sought help, but did not receive as much as needed, completed additional questions about perceived barriers to MH treatment. Twelve potential barriers were rated on 5-point scales (ranging from ‘not at all’ to ‘a lot’), covering issues such as treatment costs, distance to services, and views on treatment usefulness. For participants who endorsed the specific barrier “I prefer to manage myself,” additional questions enquired as to the main reason for this. Guided by findings from a preliminary series of principal component analyses (see Additional file 1), scores on three perceived barriers to MH treatment factors were obtained by averaging responses to the allocated items (producing scores ranging from 1 to 5 for each factor): Factor 1, ‘Structural barriers to help-seeking’ (5 items, e.g., “It is too far to travel”); Factor 2, ‘Attitudinal barriers to help-seeking’ (4 items, e.g., “I didn’t think anything could help”); and Factor 3, ‘Time commitments’ (2 items, e.g., “I am too busy caring for someone else”); see Additional file 1: Table S3 for item content and factor assignments.

#### Feasibility of internet-delivered MH services

Participants completed two questions that were used to determine whether internet-delivered MH treatments would be a feasible option for them: about their attitudes (“would you consider using the computer or the internet as a way of accessing treatment for your mental health?”); and internet access (dialup, ADSL, or broadband) in their home or elsewhere.

### Data analysis

Data were analysed using the Statistical Package for Social Sciences (SPSS version 20; Chicago, IL, USA). For overall univariate comparisons between sub-groups, simple chi-square tests (for categorical variables) or one-way ANOVAs (for continuous variables) were used, or alternatively ANCOVAs, when statistically controlling for the influence of other factors. Hierarchical logistic regressions were used in the major analyses examining relationships between potential facilitators/barriers and self-reported contacts for MH problems; Adjusted Odds Ratios (AORs) are reported, together with associated 99% Confidence Intervals (CI). The threshold for statistical significance was set at *p* < .01 for all analyses.

## Results

### Sub-groups and basic socio-demographic characteristics

There were 1,266 respondents to the ARMHS 3-year follow-up (48% of baseline respondents or 69% of those who completed at least one of the three follow-up phases), 35 of whom were excluded from the current analyses (i.e., 15 now residing in a metropolitan area and 20 with insufficient MH data). Among the 1,231 respondents with relevant data, 394 (32.0%) reported experiencing a MH problem in the last 12 months. Within this sub-sample, 143 (36.3%) indicated that they had not sought any help or advice, 176 (44.7%) reported that they had sought and received as much help as needed, and 75 (19.0%) reported receiving insufficient help. The upper portion of Table 1 presents the socio-demographic characteristics of these sub-groups.

**Table 1:** **Characteristics of Australian Rural Mental Health Study (ARMHS) participants at 3-year follow-up**

**Characteristic**	**Total sample (N = 1231)**	**No MH problems in last 12 months (N = 837)**	**Self-reported MH problems in last 12 months (N = 394)**			**Statistical comparisons (** ***p-values*** **)**	
			**Help/advice not sought (N = 143)**	**Help/advice sought**		**No** ***vs*** **. Any MH problems (df = 1)**	**Between help/advice categories (df = 2)**
				**Needs met (N = 176)**	**Needs not met (N = 75)**		
Socio-demographic variables:							
Age (mean, SD)	58.87 (13.27)	60.70 (12.95)	53.54 (13.12)	55.85 (13.10)	55.87 (12.12)	**	.243
Gender: female (n,%)	753 (61)	494 (59)	80 (56)	119 (68)	60 (80)	.024	**
Education: high school or above (n,%)	857 (70)	559 (67)	108 (76)	138 (78)	52 (69)	.002*	.309
Marital status: married/*defacto* (n,%)	950 (77)	679 (81)	103 (72)	126 (72)	42 (56)	**	.029
Remoteness (ASGC) category: remote/very remote (n,%)	268 (22)	185 (22)	26 (18)	41 (23)	16 (21)	.681	.537
Financial position: Just getting along to very poor (n,%)	362 (29)	224 (27)	43 (30)	56 (32)	39 (52)	.003*	.003*
Potential facilitators/barriers:							
Employed or volunteer work (n,%)	700 (57)	446 (53)	109 (76)	104 (59)	41 (55)	**	**
Children in house (n,%)	309 (25)	183 (22)	51 (36)	53 (30)	22 (29)	**	.493
Caring for family member/friend (n,%)	129 (11)	83 (9.9)	12 (8.4)	22 (13)	12 (16)	.347	.226
Predicted Service Need Index (mean, SD)	1.73 (2.53)	0.89 (1.54)	3.01 (2.86)	3.35 (3.11)	4.81 (3.76)	**	**
Suicidal ideation–last two-weeks (n,%)	44 (3.6)	9 (1.1)	7 (4.9)	16 (9.1)	12 (16)	**	.023
Easy access to internet (n,%)	941 (76)	628 (75)	116 (81)	144 (82)	53 (71)	.089	.111
Would use internet for MH treatment/information (n,%)	372 (30)	175 (21)	62 (43)	100 (57)	35 (47)	**	.047

Note: Separate statistical comparisons were made: 1) between those with and without self-reported mental health (MH) problems in the last 12 months; and 2) between the three help/advice categories (i.e., among those reporting problems: help/advice not sought; sought–needs met; or sought–needs not met); using either chi-square tests (for categorical variables) or one-way ANOVAs (for continuous variables): *p < 0.01; **p < 0.001.

As evidenced by the statistically significant comparisons in Table 1, on average, respondents with a self-reported 12-month MH problem were younger (55.01 *vs*. 60.70 years), more likely to have completed high school or above (76% *vs*. 67%), less likely to be married (69% *vs*. 81%), and more likely to be experiencing financial problems (35% *vs*. 27%). Within this sub-sample, the proportion of females was higher among those who had sought help, particularly those reporting unmet needs (80% female). The latter sub-group were also more likely to report financial problems (52% *vs*. 31%).

### Predicted service needs and potential facilitators/barriers

Respondents to the ARMHS 3-year survey had similar PSNI profiles to those assessed at baseline [24], namely: 67% low (0–1); 24% medium (2–5); and 9% high (>5) estimated current need for professional MH services. As detailed in the lower portion of Table 1, respondents with a self-reported 12-month MH problem had substantially higher mean PSNI scores (aggregate = 3.51 *vs*. 0.89), particularly those reporting unmet needs, whose mean score (of 4.81) was close to our threshold for ‘high predicted service need’.

The other characteristics in Table 1 were viewed as potential facilitators of and/or barriers to help-seeking, depending on your level of service need. So, for example, three-quarters (76%) of the sub-group with a 12-month MH problem who had not sought any help were currently employed or involved in volunteer work, which could have simultaneously reduced their available time for help-seeking and increased their capacity to access services (e.g., through work related connections or a better financial position). This sub-group also tended to have lower rates of suicidal ideation during the last two-weeks, relative to those seeking help (4.9% *vs*. 11.2%, *p* = .023).

As expected, those *without* a 12-month MH problem reported the lowest suicidal ideation (1.1% *vs*. 8.9% for the other three sub-groups) and they also reported the lowest preparedness to use the internet for MH treatment/information (21% *vs*. 50%), which may reflect their low current need. The remaining statistically significant associations in Table 1, showing that those *without* a 12-month MH problem had a lower likelihood of being employed or having children in the house, may simply reflect the age differentials noted earlier.

### Help-seeking patterns

At the 3-year follow-up, the overall rate of self-reported professional contacts for MH problems during the last 12 months was 18% (216 of 1,231), including 14% (170) reporting GP contacts. Non-professional contacts were reported by 16% (192), including 14% (173) reporting discussions with family/friends. Telephone or internet contacts for MH problems were also reported by 2.7% (33) of participants, most of whom (29, or 88%) also reported professional contacts. Consequently, in total, one-fifth of participants (251 of 1,231, or 20%) reported at least one contact for MH problems during the last 12 months.

As shown in Table 2, among the two sub-groups who actually reported seeking help, there were comparable patterns (i.e., no significant sub-group differences) with respect to the likelihood of having ‘any contacts’ and the estimated number of contacts (by users). Among those reporting any contacts (N = 251), the mean number was 15.15 contacts (SD = 16.45; median = 10; range: 2 to 132) during the preceding 12 months.

**Table 2:** **Self-reported** ***professional*** **,** ***non-professional*** **and** ***telephone/internet*** **contacts during the last 12 months for MH problems**

**Contact type**	**Sought help/advice for MH problems in last 12 months**						**Statistical comparisons (** ***p-values*** **)**	
	**Total sub-sample (N = 251)**		**Needs met (N = 176)**		**Needs not met (N = 75)**			
	**Any contacts N (%)**	**Number of contacts** ^**¥**^ **mean (SD)**	**Any contacts N (%)**	**Number of contacts** ^**¥**^ **mean (SD)**	**Any contacts N (%)**	**Number of contacts** ^**¥**^ **mean (SD)**	**Any contacts**	**Number of contacts** ^**¥**^
All professional contacts	216 (86)	10.35 (14.20)	154 (88)	11.03 (15.84)	62 (83)	8.66 (8.77)	.312	.269
General practitioner (GP)	170 (68)	4.32 (3.83)	122 (69)	4.31 (3.82)	48 (64)	4.35 (3.89)	.409	.948
All non-professional contacts	192 (77)	7.48 (6.76)	141 (80)	7.09 (6.15)	51 (68)	8.57 (8.19)	.038	.180
Friend or family	173 (69)	6.26 (5.33)	128 (73)	5.81 (4.84)	45 (60)	7.53 (6.41)	.046	.062
Telephone or internet contacts (e.g., Lifeline)	33 (13)	3.52 (2.06)	27 (15)	3.33 (1.47)	6 (8)	4.33 (3.88)	.115	.290
All contacts	251 (100)	15.15 (16.45)	176 (100)	15.84 (17.75)	75 (100)	13.51 (12.82)	-	.309

Note: Statistical comparisons between the sub-groups were based on chi-square tests (for categorical variables) or one-way ANOVAs (for continuous variables): *p < 0.01; **p < 0.001. ^¥^By service users (i.e., excluding those with zero contact); contact counts were derived from items with seven labelled response alternatives (ranging from ‘None’, ‘1–2 times’, to ‘13+ times’), which were assigned weights of 0, 2, 4, 6, 9, 12, and 18 contacts respectively.

The most common type of help provided was medication (47.0%), followed by counselling (37.5%), general MH information (29.1%), or help with a specific aspect of their life (21.5%), such as housing, money, or work. Comparisons between the sub-groups whose needs were met versus not met revealed similar profiles for the type of help provided: medication (50.0% *vs*. 40.0%, *p* = .146); counselling (39.8% *vs*. 32.0%, *p* = .244); general MH information (29.0% *vs*. 29.3%, *p* = .955); and help with a specific aspect of their life (22.2% *vs*. 20.0%, *p* = .703).

### Perceived barriers to adequate health care: dimensions and profiles

The three barriers dimensions that were identified (i.e., structural, attitudinal, and time commitments) provided a convenient way of representing participants’ perceptions. Nevertheless, amongst those to whom the barriers questions were addressed (N = 218), mean endorsement ratings were generally low (<2.4 on the 1–5 scale), although half (53.7%) identified some structural barriers and most (93.1%) identified some attitudinal barriers (i.e., at least one item rated above ‘not at all’).

As shown in Table 3, the sub-group reporting that their MH care needs were not met were more likely to have identified structural barriers as reasons that stopped or delayed their ability to get help. They also tended to report time commitments (*p* = .027) among their reasons for not receiving help. These comparisons were based on ANCOVAs controlling for socio-demographic differences; however, it is worth noting that across these sub-groups financial position was strongly associated with the identification of structural barriers (mean for ‘just getting along to very poor’: 2.06 *vs*. 1.39 for others, *p* < .001).

**Table 3:** **Perceived barriers and self-management profiles for selected sub-groups reporting MH problems in the last 12 months**

**Barriers to adequate health care: Factor (Potential score range: 1–5)**	**Self-reported MH problems in last 12 months**		**Statistical comparisons (** ***p-values*** **)**
	**Help/advice not sought (N = 143)**	**Help/advice sought–Needs not met (N = 75)**	
	Mean (SD)		
Structural barriers to help-seeking	1.38 (0.68)	2.15 (1.19)	**
Attitudinal barriers to help-seeking	2.30 (0.76)	2.38 (0.95)	.834
Time commitments	1.35 (0.64)	1.67 (0.95)	.027
**Self-management reasons: (Main reason for preferring to manage problems themselves)**			
	**(N = 104)**	**(N = 40)**	
	% Endorsement		
“I don’t think they know how to help”	12.5	25.0	**(df = 5)
“I’m uncomfortable talking about these problems”	35.6	15.0	
“I rely on faith and spirituality”	6.7	7.5	
“I rely on family and friends”	35.6	20.0	
“I’d be treated differently if people thought I had a mental illness”	2.9	17.5	
“I don’t think it is fair to expect it”	6.7	15.0	

Note: Other sub-groups were not asked to complete the barriers questions; statistical comparisons were based on ANCOVAs (for continuous variables), controlling for socio-demographic characteristics (using 15 dummy coded variables representing the categories detailed in Table 4), or overall chi-square tests (for categorical variables): *p < 0.01; **p < 0.001.

One of the components of attitudinal barriers (a preference for self-management) was further explored in the survey. As shown in the lower portion of Table 3, among those who did not seek help, the main reasons endorsed for self-management were feelings of discomfort talking about problems (35.6%) and reliance on family/friends (35.6%); whereas among those with unmet needs a mixture of reasons were endorsed for preferring self-management.

### Cross-sectional correlates of help-seeking for MH problems

The goal of the major multivariate analyses was to examine relationships between potential facilitators/barriers and self-reported contacts for MH problems, after taking into account the contributions of socio-demographic characteristics and predicted service needs based on recent health status indicators (e.g., symptoms/distress, adverse experiences). A series of hierarchical logistic regressions was conducted for the outcome variables of professional and non-professional service utilisation during the last 12 months, each coded as no contact (0) versus any contact (1). Socio-demographic characteristics were entered simultaneously at step 1 (see Table 4), followed by the 11 predictors listed in Table 5 at step 2.

**Table 4:** **Relationships between socio-demographic characteristics and reported** ***professional*** **and** ***non-professional*** **contacts for MH problems–ARMHS 3-year follow-up (N = 1231)**

**Socio-demographic characteristic** ^**¥**^	**Sub-group N**	**Professional service utilisation**		**Non-professional service utilisation**	
		**N (%)**	**AOR (99% CI)**	**N (%)**	**AOR (99% CI)**
Age (years):					
18–34	52	10 (19)		10 (19)	
35–44	124	31 (25)	1.74 (0.58, 5.19)	30 (24)	1.54 (0.51, 4.62)
45–54	266	60 (23)	1.51 (0.54, 4.24)	59 (22)	1.34 (0.48, 3.76)
55–64	379	63 (17)	1.10 (0.39, 3.11)	55 (15)	0.86 (0.30, 2.45)
65+	410	52 (13)	0.75 (0.26, 2.20)	40 (9.8)	0.57 (0.19, 1.71)
Gender:					
Male	478	63 (13)		48 (10)	
Female	753	153 (20)	1.64 (1.06, 2.55)*	146 (19)	2.14 (1.32, 3.45)**
Education:					
Partial schooling	303	40 (13)		32 (11)	
Completed high school or above	857	160 (19)	1.63 (0.95, 2.77)	152 (18)	1.86 (1.04, 3.33)*
Unknown	71	16 (23)	2.04 (0.85, 4.93)	10 (14)	1.50 (0.53, 4.23)
Marital status:					
Married/de facto	950	145 (15)		130 (14)	
Divorced/separated	121	31 (26)	1.67 (0.91, 3.08)	35 (29)	2.41 (1.31, 4.43)**
Widowed	90	19 (21)	1.91 (0.87, 4.19)	13 (14)	1.50 (0.62, 3.68)
Never married	70	21 (30)	2.09 (0.97, 4.49)	16 (23)	1.62 (0.71, 3.73)
ASGC category (Remoteness):					
Inner regional	544	98 (18)		89 (16)	
Outer regional	419	70 (17)	0.91 (0.57, 1.43)	63 (15)	0.92 (0.57, 1.49)
Remote	179	29 (16)	0.77 (0.41, 1.43)	23 (13)	0.67 (0.34, 1.33)
Very remote	89	19 (21)	1.13 (0.54, 2.40)	19 (21)	1.36 (0.63, 2.93)
Financial position:					
Prosperous/comfortable	198	26 (13)		28 (14)	
Reasonable	671	104 (16)	1.30 (0.70, 2.42)	91 (14)	0.99 (0.54, 1.84)
Just getting along to very poor	362	86 (24)	2.17 (1.12, 4.18)*	75 (21)	1.67 (0.86, 3.23)

Note: Based on a series of hierarchical logistic regressions, in which socio-demographic characteristics were entered simultaneously at step 1, followed by potential facilitators/barriers at step 2 (see Table 5): *p < 0.01; **p < 0.001. ARMHS: Australian Rural Mental Health Study; AOR: Adjusted Odds Ratio; CI: Confidence Interval. ^¥^Comparable categories to Table 3 of Perkins et al. (2013) [24], but based on 3-year follow-up data.

**Table 5:** **Relationships between potential facilitators/barriers and reported** ***professional*** **and** ***non-professional*** **contacts for MH problems–ARMHS 3-year follow-up**

**Characteristic**	**Sub-group N**	**Professional service utilisation**		**Non-professional service utilisation**	
		**N (%)**	**AOR (99% CI)**	**N (%)**	**AOR (99% CI)**
Employment status:					
Not currently employed	531	98 (19)		75 (14)	
Employed or volunteer work	700	118 (17)	0.83 (0.47, 1.46)	119 (17)	1.23 (0.68, 2.20)
Children in house:					
No	922	152 (17)		136 (15)	
Yes	309	64 (21)	0.76 (0.38, 1.49)	58 (19)	0.56 (0.27, 1.13)
Caring for family member/friend:					
No	1102	186 (17)		169 (15)	
Yes	129	30 (23)	0.94 (0.44, 2.05)	25 (19)	0.88 (0.41, 1.93)
Predicted Service Need Index (PSNI):					
Low (0–1)	819	73 (8.9)		66 (8.1)	
Medium (2–5)	297	76 (26)	3.29 (1.94, 5.56)**	69 (23)	3.23 (1.87, 5.59)**
High (>5)	115	67 (58)	14.00 (6.24, 31.4)**	59 (51)	12.32 (5.55, 27.3)**
Suicidal ideation (PHQ-9, item 9):					
No	1187	189 (16)		172 (15)	
Yes (present in last two-weeks)	44	27 (61)	2.41 (0.81, 7.13)	22 (50)	2.68 (0.94, 7.70)
Easy access to internet:					
No	290	50 (17)		39 (13)	
Yes	941	166 (18)	0.91 (0.48, 1.73)	155 (17)	0.85 (0.43, 1.66)
Would use internet for MH treatment/information:					
No	859	103 (12)		87 (10)	
Yes	372	113 (30)	3.13 (1.85, 5.29)**	107 (29)	3.03 (1.78, 5.17)**
Continuous variables (dummy coded)^¥^					
Structural barriers to help-seeking	(Range: 1–5)		2.30 (1.20, 4.44)**		0.88 (0.53, 1.47)
Attitudinal barriers to help-seeking	(Range: 1–5)		0.10 (0.05, 0.22)**		0.15 (0.08, 0.29)**
Time commitments	(Range: 1–5 )		1.12 (0.56, 2.25)		1.11 (0.58, 2.15)
Number of contacts with other service type	(Range: 0–42)		0.93 (0.87, 0.99)*	(0–126)	0.98 (0.95, 1.01)

Note: Based on a series of hierarchical logistic regressions, in which socio-demographic characteristics were entered simultaneously at step 1 (see Table 4), followed by the current set of predictors at step 2: *p < 0.01; **p < 0.001. ARMHS: Australian Rural Mental Health Study; AOR: Adjusted Odds Ratio; CI: Confidence Interval. ^¥^Dummy coding was used for these variables (i.e., allocating mean scores to those with “missing” data) as they only applied to selected sub-groups, namely those who answered the barriers section of the survey (i.e., MH problems but no advice sought, or needs not met) and those with at least one service contact (for the “number of contacts” variable).

As detailed in Table 4, higher rates of contact with professionals were reported by females (20% *vs*. 13%, AOR = 1.64, *p* = .004) and those in poorer financial circumstances (24% *vs*. 13%, AOR = 2.17, *p* = .003); while higher rates of contacts with non-professionals were also reported by females (19% *vs*. 10%, AOR = 2.14, *p* < .001), those who completed high school or above (18% *vs*. 11%, AOR = 1.86, *p* = .006), and divorced/separated individuals (29% *vs*. 14%, AOR = 2.41, *p* < .001). Geographical remoteness was not associated with professional or non-professional help-seeking.

Four of the predictors showed consistent patterns of association with both professional and non-professional help-seeking (see Table 5). As expected, there was essentially a dose-response relationship between predicted service need and the likelihood of using services, with a three-fold increase in the likelihood of any service contact (AORs of 3.29 and 3.23, *p* < .001) for those with a medium PSNI score, and over a twelve-fold increase (AORs of 14.00 and 12.32, *p* < .001) for those with a high PSNI score. Although statistically non-significant, recent suicidal ideation (AORs of 2.41, *p* = .037, and 2.68, *p* = .016) was consistently associated with a higher likelihood of service use (over and above the influences captured in the PSNI), as was preparedness to use the internet for MH treatment/information (AORs of 3.13 and 3.03, *p* < .001). Conversely, after controlling for the other associations, higher endorsement of attitudinal barriers (by those to whom these questions were addressed) was associated with a substantial reduction in the likelihood of seeking any help, either professional or non-professional (AORs of 0.10 and 0.15, *p* < .001).

Two additional statistically significant associations with professional service usage were detected. Firstly, higher endorsement of structural barriers was associated with an increased likelihood of reporting professional contacts (AOR of 2.30, *p* < .001). Secondly, there was a tendency for (each unit of) increased contacts with non-professionals to reduce the likelihood of accessing professional help (AOR of 0.93, *p* = .003).

**Figure 1:**
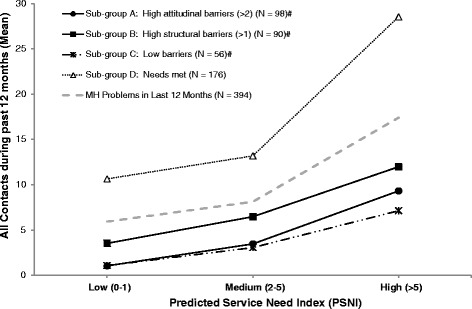
**Self-reported contacts by individuals reporting mental health problems during the past 12 months: selected subgroups by predicted service need.** (# Among those asked the barriers questions-i.e., advice not sought, or needs not met).

Finally, to better understand relationships between predicted service needs, barriers and the overall number of contacts for MH problems in the last 12 months (N = 394), we plotted mean contact patterns for selected sub-groups (see Figure 1). These sub-groups were selected for illustrative purposes, based on the key predictors identified in the major regression analyses (Table 5); it should also be noted that, where appropriate, those who did not seek advice are included in these sub-groups (with zero service usage), as they also identified potential barriers. Three broad patterns are evident: increased service need (PSNI) is clearly associated with increased contacts for all sub-groups; individuals reporting their needs were met had the highest overall contact rates; and ‘barriers’ are likely to be associated with a mixed pattern of help-seeking–potentially impacting differentially on the likelihood of seeing someone and the number of contacts by those who actually sought help.

## Discussion

### Brief summary of selected findings

This brief summary is provided, to help frame subsequent discussion:

1. one-third (32.0%) of the total sample self-reported MH problems in the last 12 months, amongst whom one-third (36.3%) did not seek any help and one-fifth (19.0%) indicated their needs were not met;
2. overall help-seeking rates were similar to baseline findings (professionals: 17.5% *vs*. 17.3%; non-professionals: 15.6% *vs*. 11.6%) [24], rising to approximately half of the sub-group in the highest need category (professionals: 58.3%; non-professionals: 51.3%);
3. observed help-seeking rates and the dose-response relationship between predicted service needs (indicative of the severity of current problems) and the likelihood of using services were also broadly consistent with national survey findings in Australia [25] and elsewhere [23]; and
4. attitudinal barriers were consistently associated with a lower likelihood of help seeking, even after socio-demographic factors, predicted service needs, and structural barriers were taken into account.

### Unravelling needs, help-seeking and barriers

Interestingly, few characteristics distinguished between participants who did and did not seek help, or those who reported receiving enough help. While females were more likely to seek help, they were also over-represented among those with unmet needs. Financial difficulties were also more marked among those reporting unmet needs, a common finding in many studies [34,35], while conversely employed participants with MH problems were less likely to seek help. PSNI scores were higher among help-seekers, and highest in the group who did not feel they had received enough help; however, participants who had received enough help may have initially had higher PSNI scores, but these reduced as a result of effective assistance. Notwithstanding, severity was highest among people who were unable to access sufficient help (despite attempts to do so), which is reflective of the importance of providing accessible and acceptable services in rural areas.

As indicated by our principal component analysis, perceived barriers to mental health care fell under the domains of structural (e.g., costs, distance), attitudinal (e.g., stigma concerns, confidentiality), and time commitments. At first glance, the barriers-related findings in Table 4 and Figure 1 appear somewhat inconsistent; however, the logistic regressions examined predictors of the likelihood of seeking *any* professional or non-professional help, while Figure 1 details the mean number of contacts by those reporting MH problems. The most parsimonious explanation is that barriers play a mixture of roles (and/or come in a variety of forms, complicating their measurement). For example, attitudinal barriers may contribute both to a lower likelihood of seeking any help (e.g., privacy concerns, or limited possibilities) and to lower contact rates even when help is initially sought (e.g., self-management preferences). On the other hand, the identification of structural barriers may arise within the context of help-seeking (e.g., high costs or transport issues), by those with current needs (and, in the current study, poorer financial circumstances), and contribute to a lower number of contacts than considered desirable or optimal–resulting in self-reported unmet needs. Additionally, other factors, such as rural stoicism, could contribute both to not seeking help and to the non-identification of barriers. It should also be acknowledged that higher self-management preferences should not be automatically viewed negatively, since many associated strategies (e.g., reducing substance use, increasing participation and physical activity) are endorsed by the community as a positive influence on mental health [36].

The present study did not seek to examine or disentangle the potential drivers of current MH problems and/or barriers to care, but rather to assess their contributions to professional and non-professional help-seeking. However, public health or other interventions designed to improve or optimise the provision of MH care should ideally target factors that both reduce the need for care and minimise potential barriers for those seeking help. Three factors from our study that appear to have relevance to each of these are relationships (support networks), financial difficulties and stigma. For example, being married was associated with a lower likelihood of experiencing MH problems (reinforcing our earlier findings [37]), and with lower help-seeking, while recent financial problems was associated with higher rates of both. Attitudinal factors, including perceived stigma, can also contribute to higher distress and lower help seeking. Likewise, interventions that seek to reduce suicidal ideation and behaviours and/or increase associated help-seeking can impact on overall MH care profiles.

That attempting to seek help is an important contributor to the recognition of structural barriers concords with recent research demonstrating structural barriers are identified more as severity increases [23]. Structural barriers may not stop rural residents from seeking help initially, however, they do impact on their ability to receive an adequate level of care; and, more generally, they impact on treatment drop-out [23]. Perhaps not surprisingly, structural barriers did not have a significant association with non-professional help-seeking, indicating that challenges to accessing services may not influence rural residents’ desire or ability to receive some assistance; likewise, geographical remoteness did not differentially affect help-seeking. On the other hand, attitudinal barriers reduce the likelihood of help-seeking substantially.

### Study limitations

The present study has several limitations, including under-representation of younger adults and reliance on self-reported MH data; the target group for the barriers questions could have also been expanded to include those who reported their needs were met. Perceived barriers may also vary across cultures [38], MH problems and treatment providers [39], and possibly with increasing comorbidity, although this has yet to be established [34]; none of which were assessed in the current study. The broader health service context within which the ARMHS project was conducted also sets some limits on international comparisons, since Australia has a centralised health system, with subsidised access to General Practitioners and medical specialists. Conversely, the study also has considerable strengths, such as community (as opposed to service) based recruitment, high representation of participants from remote/very remote regions, and applicability of our findings to rural areas. While the current study focussed on mental health problems, it may also be the case that older adults in rural areas experience a constellation of barriers to health care [35], almost regardless of the nature of their problems.

## Conclusions

This paper explored cross-sectional associations between predicted service needs, perceived barriers, and professional and non-professional help-seeking by rural and remote residents in NSW, Australia. Previous research has shown that rural residents may have a preference for informal help-seeking and consider professional service use as a last resort [15]. However, our findings about attitudinal barriers apply to both professional and non-professional help-seeking. The implications of this may be considerable in relation to the initiation of public health interventions and optimising rural MH service use. In recent years, there has been an emphasis on increasing the provision of services through innovative strategies, such as internet-delivered treatments [40] and information [20], telehealth facilities, and attempts to attract more MH professionals to rural and remote areas of Australia [41]. However, targeting issues related to physical availability alone may not be sufficient to adequately increase service use by rural and remote residents. In addition, although internet-delivered treatments may increase service contacts by current help-seekers, they may not necessarily increasing overall help-seeking.

Participants with recent suicidal ideation were generally more likely to report help-seeking in the past 12 months, however, the relevant sample sizes were small and, within the context of the multivariate analyses, these effects were not statistically significant. Research indicates that a contributor to elevated rural suicide rates may be the lower help-seeking behaviour in non-metropolitan areas [42]. The present findings do not indicate a lack of desire for help; moreover, among a younger sample rates of and barriers to formal service use for those experiencing suicidal thoughts may have been different.

Preparedness to use the internet was higher among those reporting MH problems and other service contacts, which is consistent with earlier findings [26,43]. However, while internet-delivered treatments may eventually improve rural residents’ access to MH resources [20], at present they are subject to many of the same barriers as traditional MH services. Addressing perceptions of mental illness and attitudes towards help-seeking, through the development and evaluation of appropriate interventions, is likely to be vital in improving and optimising service use and MH outcomes in rural and remote Australia.

## Additional file

Additional file 1: Table S1. Relationships between current health status characteristics and reported professional contacts for MH problems – ARMHS baseline and 3-year follow-up. **Table S2.** Relationships between current health status characteristics and reported non-professional contacts for MH problems – ARMHS baseline and 3-year follow-up. **Table S3.** Perceived barriers and self-management profiles for selected sub-groups reporting MH problems in the last 12 months. **Table S4.** Relationships between socio-demographic characteristics and reported professional and non-professional contacts for MH problems – ARMHS 3-year follow-up (N = 1231).

## Abbreviations

ADSL: Asymmetric digital subscriber line
ANCOVA: Analysis of covariance
ANOVA: Analysis of variance
AOR: Adjusted odds ratio
ARMHS: Australian rural mental health study
AUDIT: Alcohol use disorders identification test
ASGC: Australian standard geographic classification
CI: Confidence Interval
GP: General practitioner
K10: Kessler-10 measure of current psychological distress
MH: Mental health
PHQ-9: Patient Health Questionnaire-9
PSNI: Predicted (Provisional) service need index
SD: Standard deviation
