# Supplementary material for: Self-reported contacts for mental health problems by rural residents: predicted service needs, facilitators and barriers
Source: BMC Psychiatry. 2014 Sep 6;14:249. doi: 10.1186/s12888-014-0249-0 (PMC4172961; doi:10.1186/s12888-014-0249-0)
Supplement: Additional file 1: Table S1. — Relationships between current health status characteristics and reported professional contacts for MH problems – ARMHS baseline and 3-year follow-up. Table S2. Relationships between current health status characteristics and reported non-professional contacts for MH problems – ARMHS baseline and 3-year follow-up. Table S3. Perceived barriers and self-management profiles for selected sub-groups reporting MH problems in the last 12 months. Table S4. Relationships between socio-demographic characteristics and reported professional and non-professional contacts for MH problems – ARMHS 3-year follow-up (N = 1231). [file 12888_2014_249_MOESM1_ESM.docx]

**“Self-reported contacts for mental health problems by rural residents: predicted service needs, facilitators and barriers” (*Handley et al.*)**

**Supplementary Tables** (July, 2014)

This supplementary document briefly addresses two issues of direct relevance to the major analyses in the current paper:

1) The development and confirmation of our Predicted Service Need Index (PSNI); and

2) The identification of barriers dimensions and derivation of associated scores.

**Development and confirmation of Predicted Service Need Index (PSNI)**

In the absence of detailed questions in the ARMHS surveys about met and unmet needs for specific professional services, we developed the Provisional Service Need Index (PSNI) using the baseline survey data, to quantify each individuals likely current need for services; see Perkins et al. (2013) for further details [[1](#_ENREF_1)].

The PSNI assigns integer weights (from 0 to 3) to 16 categories across seven health status measures: overall ratings of mental and physical health, using 5-point scales from ‘poor’ to ‘excellent’; Kessler-10 (K10) [[2](#_ENREF_2), [3](#_ENREF_3)]: current psychological distress; Alcohol Use Disorders Identification Test (AUDIT) [[4](#_ENREF_4), [5](#_ENREF_5)]: current hazardous alcohol use; Patient Health Questionnaire-9 (PHQ-9) [[6](#_ENREF_6)]: current depressive symptoms; recent adverse life events; and current smoking status. See the left-hand column of Table S1 for the health status categories and weights.

Overall scores on the PSNI range from 0 to 14 (obtained by simple summation of the category weights). Based on a Receiver Operating Characteristic (ROC) analysis using the ARMHS baseline data (N = 2,150), and other distributional considerations, cut-off points were also identified for the PSNI: *low* (0-1); *medium* (2-5); and *high* (>5) estimated current need for professional services [[1](#_ENREF_1)].

Before undertaking the major analyses for the current paper, we used the 3-year ARMHS follow-up data to briefly review the PSNI’s performance. Once again, a series of hierarchical logistic regressions was used, with socio-demographic characteristics entered simultaneously at step 1, followed by each of the selected health status indicators separately at step 2. The binary outcome measures in these analyses were self-reported professional and non-professional service utilisation during the last 12 months, each coded as no contact (0) versus any contact (1). The purpose of these preliminary analyses was simply to confirm whether or not the categorical health status indicators showed similar relationships to the outcome measures as those found at baseline – using a different but parallel dataset (i.e., the same predictors and outcome measures, but re-assessed three years later).

The left-hand columns of Tables S1 and S2 reproduce the baseline findings for professional and non-professional contacts respectively (N = 2,101) [[1](#_ENREF_1)], while the right-hand columns present the corresponding findings from separate analyses based solely on 3-year follow-up data (N = 1,231).

In addition to the above, we conducted a corresponding series of combined analyses, using generalised linear models (and generalised estimating equations, in particular), which included both the baseline and 3-year service contact data and predictors, and appropriate phase and within subject codes (Participant ID, N = 2,218 unique individuals, with complete socio-demographic data). There were no significant phase by health status indicator interactions in any of these analyses for the outcomes of self-reported professional and non-professional service utilisation during the preceding 12 months.

The various analyses describe above produced substantially consistent findings, from both the within phase analyses and the combined analyses. That is, the available evidence suggests that the cross-sectional relationships between the selected set of health status indicators and the likelihood of reporting concurrent service use were maintained across phases; (although individuals could clearly change their health status over time and/or their patterns of help-seeking – which were not the focus of the current analyses).

Since the basis on which the original baseline PSNI category weights were assigned (i.e., the strength of associations with reported professional service use) [[1](#_ENREF_1)] was confirmed in the 3-year ARMHS data, we recommend continued use of these category weights in subsequent ARMHS phases and analyses. Confirmation of the overall integrity of the PSNI also prompted us to relabel it as a “Predicted Service Need Index” (as opposed to “Provisional ...” [[1](#_ENREF_1)]).

Finally, while the two sets of analyses reported in Tables S1 and S2 (i.e., for the baseline, and 3-year follow-up datasets) were discrete, they involved substantially the same participants. The correlation between PSNI scores at baseline and 3-year follow-up was 0.60 (*p* < .001), with similar distributions on each occasion: *low* (0-1), 65% *vs*. 67%; *medium* (2-5), 24% *vs*. 24%; *high* (>5), 11% *vs*. 9%; approximately two-thirds of participants (68%) fell within the same broad category on these two occasions (Kappa = 0.35, *p* < .001).

**Table S1 Relationships between current health status characteristics and reported *professional* contacts for MH problems – ARMHS baseline and 3-year follow-up**

| **Health status characteristic** | **ARMHS Baseline (N = 2101)^¥^** | | |  | **ARMHS 3-year follow-up (N = 1231)** | | |
| --- | --- | --- | --- | --- | --- | --- | --- |
|  | **Sub-group**  **N** | **Professional service utilisation** | |  | **Sub-group**  **N** | **Professional service utilisation** | |
|  |  | **N (%)** | **AOR (99%CI)** |  |  | **N (%)** | **AOR (99%CI)** |
| Overall mental health: |  |  |  |  |  |  |  |
| Good to excellent [0] | 1825 | 223 (12) |  |  | 1060 | 128 (12) |  |
| Poor / fair [3] | 273 | 140 (51) | 6.49 (4.47, 9.43)** |  | 160 | 85 (53) | 7.22 (4.38, 11.9)** |
| Overall physical health: |  |  |  |  |  |  |  |
| Good to excellent [0] | 1671 | 242 (14) |  |  | 920 | 121 (13) |  |
| Poor / fair [1] | 426 | 121 (28) | 2.25 (1.59, 3.18)** |  | 92 | 121 (31) | 2.67 (1.73, 4.14)** |
| Kessler K-10 (current distress) score: |  |  |  |  |  |  |  |
| 10-15 [0] | 1481 | 145 (9.8) |  |  | 947 | 97 (10) |  |
| 16-24 [1] | 478 | 139 (29) | 3.43 (2.41, 4.88)** |  | 208 | 79 (38) | 4.69 (2.90, 7.61)** |
| 25-50 [3] | 131 | 77 (59) | 11.5 (6.80, 19.5)** |  | 59 | 35 (59) | 10.0 (4.60, 21.9)** |
| AUDIT: |  |  |  |  |  |  |  |
| 0-7 [0] | 1785 | 294 (16) |  |  | 1060 | 177 (17) |  |
| 8+ [1] | 294 | 65 (22) | 1.40 (0.91, 2.14) |  | 160 | 35 (22) | 1.63 (0.91, 2.93) |
| PHQ-9 symptomatology: |  |  |  |  |  |  |  |
| No symptoms [0] | 1686 | 213 (13) |  |  | 971 | 121 (13) |  |
| Depressive symptoms [2] | 400 | 147 (37) | 3.62 (2.58, 5.07)** |  | 235 | 89 (38) | 3.56 (2.67, 5.58)** |
| Recent adverse life events: |  |  |  |  |  |  |  |
| 0-2 [0] | 1644 | 223 (14) |  |  | 1027 | 144 (14) |  |
| 3-5 [1] | 359 | 96 (27) | 2.12 (1.47, 3.06)** |  | 167 | 61 (37) | 2.86 (1.74, 4.69)** |
| >5 [3] | 46 | 29 (63) | 7.74 (3.36, 17.8)** |  | 12 | 6 (50) | 3.79 (0.79, 18.1) |
| Smoking status: |  |  |  |  |  |  |  |
| No [0] | 1820 | 283 (16) |  |  | 1093 | 184 (17) |  |
| Yes [1] | 265 | 78 (29) | 1.95 (1.31, 2.91)** |  | 121 | 28 (23) | 1.11 (0.59, 2.10) |

*Note*: Based on a series of hierarchical logistic regressions, in which socio-demographic characteristics were entered simultaneously at step 1 (age, gender, education, marital status, geographical remoteness, and perceived financial position), followed by each of the current health status variables separately at step 2: * *p* < 0.01; ** *p* < 0.001. Only participants with the full set of socio-demographic characteristics were included. ARMHS: Australian Rural Mental Health Study; AOR: Adjusted Odds Ratio; CI: Confidence Interval. Values in square brackets in the left-hand column reflect the weight given to each response category in calculating a Predicted Service Need Index (PSNI). ^¥^ As reported in Table 4 of Perkins et al. (2013) [[1](#_ENREF_1)].

**Table S2 Relationships between current health status characteristics and reported non-*professional* contacts for MH problems – ARMHS baseline and 3-year follow-up**

| **Health status characteristic** | **ARMHS Baseline (N = 2101)^¥^** | | |  | **ARMHS 3-year follow-up (N = 1231)** | | |
| --- | --- | --- | --- | --- | --- | --- | --- |
|  | **Sub-group**  **N** | **Non-professional service utilisation** | |  | **Sub-group**  **N** | **Non-professional service utilisation** | |
|  |  | **N (%)** | **AOR (99%CI)** |  |  | **N (%)** | **AOR (99%CI)** |
| Overall mental health: |  |  |  |  |  |  |  |
| Good to excellent [0] | 1825 | 145 (7.9) |  |  | 1060 | 110 (10) |  |
| Poor / fair [3] | 273 | 95 (35) | 5.55 (3.64, 8.45)** |  | 160 | 80 (50) | 7.75 (4.61, 13.0)** |
| Overall physical health: |  |  |  |  |  |  |  |
| Good to excellent [0] | 1671 | 161 (9.6) |  |  | 920 | 112 (12) |  |
| Poor / fair [1] | 426 | 79 (19) | 2.33 (1.54, 3.52)** |  | 92 | 78 (26) | 2.40 (1.51, 3.82)** |
| Kessler K-10 (current distress) score: |  |  |  |  |  |  |  |
| 10-15 [0] | 1481 | 89 (6.0) |  |  | 947 | 86 (9.1) |  |
| 16-24 [1] | 478 | 98 (21) | 3.61 (2.37, 5.49)** |  | 208 | 70 (34) | 4.44 (2.67, 7.39)** |
| 25-50 [3] | 131 | 54 (41) | 10.4 (5.87, 18.5)** |  | 59 | 32 (54) | 9.16 (4.11, 20.4)** |
| AUDIT: |  |  |  |  |  |  |  |
| 0-7 [0] | 1785 | 191 (11) |  |  | 1060 | 158 (15) |  |
| 8+ [1] | 294 | 47 (16) | 1.70 (1.03, 2.78)* |  | 160 | 32 (20) | 1.75 (0.94, 3.24) |
| PHQ-9 symptomatology: |  |  |  |  |  |  |  |
| No symptoms [0] | 1686 | 137 (8.1) |  |  | 971 | 114 (12) |  |
| Depressive symptoms [2] | 400 | 104 (26) | 3.77 (2.54, 5.58)** |  | 235 | 73 (31) | 2.74 (1.70, 4.42)** |
| Recent adverse life events: |  |  |  |  |  |  |  |
| 0-2 [0] | 1644 | 131 (8.0) |  |  | 1027 | 129 (13) |  |
| 3-5 [1] | 359 | 80 (22) | 3.06 (2.02, 4.64)** |  | 167 | 54 (32) | 2.63 (1.56, 4.43)** |
| >5 [3] | 46 | 19 (41) | 5.63 (2.40, 13.2)** |  | 12 | 5 (42) | 2.60 (0.50, 13.5) |
| Smoking status: |  |  |  |  |  |  |  |
| No [0] | 1820 | 191 (10) |  |  | 1093 | 164 (15) |  |
| Yes [1] | 265 | 47 (18) | 1.53 (0.95, 2.48) |  | 121 | 27 (22) | 1.17 (0.61, 2.25) |

*Note*: Based on a series of hierarchical logistic regressions, in which socio-demographic characteristics were entered simultaneously at step 1 (age, gender, education, marital status, geographical remoteness, and perceived financial position), followed by each of the current health status variables separately at step 2: * *p* < 0.01; ** *p* < 0.001. Only participants with the full set of socio-demographic characteristics were included. ARMHS: Australian Rural Mental Health Study; AOR: Adjusted Odds Ratio; CI: Confidence Interval. Values in square brackets in the left-hand column reflect the weight given to each response category in calculating a Predicted Service Need Index (PSNI). ^¥^ As reported in Table 4 of Perkins et al. (2013) [[1](#_ENREF_1)].

**Identification of barriers dimensions**

Separate sets of questions about barriers to mental health care and physical health care were included in the ARMHS 3-year follow-up survey, enquiring about “... reasons that might have stopped or delayed your ability to get help” ... “when you needed it” or “... from getting as much help as you thought you needed”. In each case, 12 potential barriers items were rated on 5-point scales (ranging from ‘not at all’ to ‘a lot’), covering issues such as treatment costs, distance to services, and views on treatment usefulness (see Table S3 for item content).

Unfortunately, the ‘skip routines’ within the ARMHS survey instructed only two sub-groups (N = 218) to complete the 12 ‘perceived barriers (to MH care)’ questions (i.e., those with a 12-month MH problem: who did not seek any help; or whose needs were not met); however, 248 complete sets of responses were available, as some respondents whose needs were met also completed these questions. To facilitate a more comprehensive examination of the underlying barriers dimensions, these data were pooled with responses to the corresponding 12 questions about ‘perceived barriers (to physical health care)’ from respondents who self-reported poor/fair physical health in the past 12 months (N = 284); this pooled set of 532 responses came from 445 participants.

It should be noted that in pooling these data no assumptions were made about the overall conceptualisation of treatment barriers. More generally, it may well be the case that barriers to mental and physical health care differ somewhat from each another, either in their nature or applicability, or the extent to which they are influenced by other personal, cultural or contextual factors. However, to the extent that we gave participants the same fixed list of potential barriers to rate (for both mental and physical health care), it may also be the case that the associations amongst these items are broadly similar. Moreover, we undertook the analyses reported below within both the individual and pooled barriers datasets.

Following a preliminary examination of item distributions and associations, one item was dropped from subsequent analyses (“I asked but didn’t get the help”); for which 83% answered ‘Not at all’, and which had only 12% shared variance with the other items. Table S3 reports factor loadings from a T-technique (i.e., total variation) principal component analysis of the perceived barriers items (N = 532 sets of ratings), using an oblique rotation (correlations among these factors ranged from 0.23 to 0.25). A three-factor solution accounted for 60.6% of the item variance. Comparable patterns emerged from parallel analyses of the separate sets of mental and physical health care items (accounting for 60.0% and 60.8% of the variance, respectively).

Scores on the perceived barriers factors were obtained by averaging responses to the allocated items (see Table S3), ***separately*** for mental and physical health care (producing scores ranging from 1 to 5): Factor 1, ‘Structural barriers to help-seeking’ (5 items); Factor 2, ‘Attitudinal barriers to help-seeking’ (4 items); and Factor 3, ‘Time commitments’ (2 items).

**Table S3 Principal component analysis of perceived barriers to adequate health care – oblique rotation (N = 532 sets of responses)** ^¥^

| **Factor** | **Survey item** | **Factor loadings** | | |
| --- | --- | --- | --- | --- |
|  |  | **1** | **2** | **3** |
| Structural barriers to help-seeking | It costs too much to get there | **.931** | -.029 | .018 |
|  | It is too far to travel | **.885** | .012 | .040 |
|  | Getting transport there is hard for me | **.839** | -.038 | -.162 |
|  | I couldn't afford to pay for the service | **.664** | .186 | -.024 |
|  | It takes too long to get an appointment | **.613** | -.055 | .278 |
|  |  |  |  |  |
| Attitudinal barriers to help-seeking | I was afraid to ask for help, or what others would think of me if I did | -.016 | **.740** | .103 |
|  | I didn't think anything could help | .102 | **.708** | -.027 |
|  | I prefer to manage myself | -.118 | **.690** | -.058 |
|  | I feel like my problems won't stay private | .125 | **.664** | .069 |
|  |  |  |  |  |
| Time commitments | I can't get time away from work | .000 | .060 | **.772** |
|  | I am too busy caring for someone else | -.011 | -.003 | **.772** |
|  | Eigenvalue | 3.83 | 1.76 | 1.07 |
|  | % of total variance | 34.82 | 16.02 | 9.75 |

^¥^ Based on responses to separate sets of questions about physical health care needs and MH problems, enquiring about “... reasons that might have stopped or delayed your ability to get help” ... “when you needed it” or “... from getting as much help as you thought you needed”; respondents included those with self-reported poor/fair physical health (N = 284) or MH problems in the past 12 months (N = 248); loadings of .50 or higher are set in bold font.

**References**

1. Perkins D, Fuller J, Kelly BJ, Lewin TJ, Fitzgerald M, Coleman C, Inder KJ, Allan J, Arya D, Roberts R, Buss R: **Factors associated with reported service use for mental health problems by residents of rural and remote communities: cross-sectional findings from a baseline survey.** *BMC Health Serv Res* 2013, **13**.

2. Andrews G, Slade T: **Interpreting scores on the Kessler psychological distress scale (K10).** *Aust N Z J Pub Health* 2001, **25:**494-497.

3. Kessler RC, Barker PR, Colpe LJ, Epstein JF, Gfroerer JC, Hiripi E, Howes MJ, Normand SLT, Manderscheid RW, Walters EE, Zaslavsky AM: **Screening for serious mental illness in the general population.** *Arch Gen Psychiatry* 2003, **60:**184-189.

4. Saunders JB, Aasland OG, Babor TF, de la Fuente JR, Grant M: **Development of the Alcohol Use Disorders Identification Test (AUDIT): WHO collaborative project on early detection of persons with harmful alcohol consumption - II.** *Addiction* 1993, **88:**791-804.

5. Conigrave KM, Hall WD, Saunders JB: **The AUDIT questionnaire: choosing a cut-off score.** *Addiction* 1995, **90:**1349-1356.

6. Kroenke K, Spitzer RL, Williams JB: **The PHQ-9: validity of a brief depression severity measure.** *J Gen Intern Med* 2001, **16:**606-613.
